# Decompression Surgery of Orbital Compartment Syndrome—Analysis of Surgery Procedures and Visual Function

**DOI:** 10.3390/jcm14103453

**Published:** 2025-05-15

**Authors:** Alexander Kilgue, Christoph Pfeiffer, Lars-Uwe Scholtz, Conrad Riemann, Annika Hoyer, Maged Alnawaiseh, Ingo Todt

**Affiliations:** 1 Department of Otolaryngology, Head and Neck Surgery, Medical School OWL, Bielefeld University, Campus Klinikum Bielefeld Mitte, Teutoburgerstr. 50, 33604 Bielefeld, Germany; alexander.kilgue@klinikumbielefeld.de (A.K.); christoph.pfeiffer@klinikumbielefeld.de (C.P.); lars-uwe.scholtz@klinikumbielefeld.de (L.-U.S.); conrad.riemann@klinikumbielefeld.de (C.R.); 2 Biostatistics and Medical Biometry, Medical School OWL, Bielefeld University, 33604 Bielefeld, Germany; annika.hoyer@uni-bielefeld.de; 3 Department of Ophthalmology, Medical School OWL, Bielefeld University, Campus Klinikum Bielefeld Mitte, 33604 Bielefeld, Germany; maged.alnawaiseh@klinikumbielefeld.de

**Keywords:** orbital compartment syndrome, retrobulbar hemorrhage, orbital decompression, trauma

## Abstract

**Objective**: Various orbital conditions (trauma, autoimmune thyroid disease, tumors, infections, congenital malformations) may lead to a consecutive increase in orbital cavity pressure resulting in orbital compartment syndrome (OCS). OCS is associated with acute loss of visual function and a high risk of permanent damage to the optic nerve (compressive optic neuropathy). Orbital decompression surgery (ODS) is a time-critical procedure that reduces pressure on the optic nerve, thereby improving visual function. The surgical management protocol for orbital decompression is not standardized and varies. Surgical techniques differ in orbital fat decompression, lateral canthotomy, and decompression of the medial orbital wall and floor. This retrospective study aims to evaluate surgery procedures and the outcome of visual function after orbital decompression surgery. **Methods**: In this retrospective study, we evaluated 28 patients (17 male, 11 female) with orbital compartment syndrome from May 2016 to October 2024. All patients underwent orbital decompression surgery as first-line treatment. Visual acuity (VA), diplopia, and ocular motility were analyzed pre- and postoperatively. Recovery was defined as postoperative improvement of vision, diplopia, and ocular motility. Linear and logistic regression analyses were used to assess the associations between clinically relevant risk factors and primary outcomes. **Results**: Orbital decompression surgery was performed with a median of 8.40 h (Q1: 4.80, Q3: 24.00) upon occurrence of symptoms. The average preoperative measured VA (logMAR) of the affected eye was 1.0. A total of 46% of the patients were preoperatively categorized as ”blind“ according to the WHO visual impairment categories. A total of 96% of the patients showed preoperative ocular motility impairment. Diplopia was preoperatively present in 46% of the patients. After orbital decompression surgery, postoperative visual acuity improved in 36% of the patients. Ocular motility improved by 67% and diplopia by 62% after ODS. The primary surgery technique was two-wall decompression in 68% (19/28) of the cases, followed by one-wall decompression (21%; 6/28), and three-wall decompression (11%; 3/28). Lateral decompression (82%; 23/28) and medial wall decompression (93%; 26/28) were the primary procedures performed. Orbital floor wall decompression was performed in only 14% (4/28) of the cases. Regression analysis revealed a statistically significant effect of preoperative measured vision on postoperative vision, while accounting for age, sex, and time to surgery. **Conclusions**: Orbital decompression surgery is the time-sensitive first-line treatment of acute visual function loss in OCS. Our data showed a postoperative improvement in visual acuity in 36% of the patients, along with considerable improvement rates in diplopia and ocular motility. The primary surgery technique was a two-wall decompression approach with lateral wall decompression and medial wall decompression. Center-specific timeline optimization of OCS patients is essential.

## 1. Introduction

The orbit is a relatively rigid bone construct with limited adaptability to changes in orbital cavity pressure and, therefore, a high risk of orbital compartment syndrome (OCS) with subsequent optic nerve ischemia. OCS was first described in 1950 by Gordan and McCrae [1]. Swelling or bleeding behind the eyeball (bulbus oculi) may result in impaired vision, proptosis, diplopia, and ocular motility. Visual loss, specifically blindness (amaurosis), is a severe complication of OCS resulting from persistent ischemia of the optic nerve [2].

Retrobulbar hemorrhage (RBH) secondary to orbito-facial trauma is a relatively rare but severe pattern that may lead to orbital compartment syndrome. The risk of RBH in facial trauma with or without bone fractures is less than one percent [3]. Iatrogenic ophthalmic surgery and non-ophthalmic procedures (such as sinus surgery and craniofacial surgery) are well reported as causes of RBH [4]. There is a wide range of etiologies which can lead to retrobulbar hemorrhage and/or an increase in orbital pressure [5].

Vision, diplopia, and ocular motility can be affected by of OCS and RBH. Detection and therapy of OCS and RBH is time critical as it may lead to permanent blindness within hours. The distinction between OCS and RBH is relevant, as urgent ODS can be necessary under certain circumstances, and decisions may be based solely on clinical signs. Bleeding of RBH can be intraconal and extraconal, which affects the clinical outcomes of the chosen surgical approach [6]. Orbital compartment syndrome and retrobulbar hemorrhage are considered a clinical diagnosis, often based on clinical examination and the patient’s history upon presentation. Clinical signs of RBH include impaired visual acuity, ocular immobility, diplopia, hard eyeball, pain, proptosis, and a lost light reflex.

Several authors have recommended orbital decompression surgery within 2 h in case of retrobulbar hemorrhage, along with the results from Hayreh and Weingeist (1980) for animal studies of irreversible damage of the optic nerve due to ischemia after 105 min [7]. Irreversible optic ischemia may occur within the first hour, while permanent visual loss is expected within a range of 1.5–2 h upon the occurrence of OCS symptoms. The earlier treatment begins, the better the patient’s visual recovery is expected to be [8].

Although preoperative radiological imaging (CT, MRI of the head and/or orbit) is beneficial, treatment should not be delayed solely to obtain radiological imaging to preserve the patient’s eyesight, according to Edmunds et al. (2019) [9]. The need for radiological imaging often depends on the severity of visual impairment. It may be redundant not to delay treatment, as an OCS diagnosis can be based on clinical signs and symptoms. In case of required further decompression surgery after initial ODS, additional imaging can be helpful to identify vascular malformations, debris, or anatomical status quo.

Due to its time-critical nature, the initial assessment for orbital compartment syndrome typically occurs upon the patient’s arrival in the emergency department (ED) by an ED physician, often without specialized training in ophthalmology [9]. A complete and standardized examination by an ophthalmologist might not be possible at the time of the patient’s presentation in the emergency department due to the time-critical aspect of OCS.

A clinical examination by an ED physician may be limited to generally subjective observations of bulbar proptosis, a hardened orbital bulb, a history of pain, vision impairment, pupillary deficit, or diplopia. To make it even more complicated, the assessment of ocular motility or pupils can be misleading in cases of unconscious patients, alcohol and drug intoxication, or traumatic mydriasis [10].

Orbital decompression surgery aims to reduce the pressure on and reestablish perfusion of the optic nerve. ODS consists of several techniques often combined depending on the etiology of OCS and the severity of visual impairment. The surgical management protocol for orbital decompression is not standardized and varies [11].

As an effective method of orbital decompression surgery, lateral canthotomy (LC) and inferior cantholysis (IC) are established. LC/IC is a standard first-line emergency decompression method usually performed under local anesthesia at the patient’s bedside. LC/IC can immediately reduce the intra-orbital pressure.

Further standard ODS procedures include techniques performed under general anesthesia, such as lateral wall decompression, orbital floor decompression, endonasal medial wall decompression (after partial removal of the lamina papyracea), and orbital fat decompression. Often, surgical decompression techniques are combined depending on the etiology of OCS.

This retrospective study aims to analyze the visual function outcome after orbital decompression surgery. This study evaluates surgical outcomes to optimize clinical decision-making in cases of orbital compartment syndrome.

## 2. Materials and Methods

We retrospectively analyzed 28 patients undergoing orbital decompression surgery due to orbital compartment syndrome (OCS) and retrobulbar hemorrhage at the Department of Otolaryngology, Head and Neck Surgery, Medical School OWL, Bielefeld University, Campus Klinikum Bielefeld Mitte, Germany, from May 2016 to October 2024.

Key data collected included etiology, the time between symptom occurrence and surgery, surgical techniques, preoperative symptoms, and postoperative outcomes after OCS. Postoperative outcomes, including ocular motility (improvement or persistence of motility impairment), visual acuity (changes in pre- and postoperative visual acuity), and diplopia (presence or absence of double vision after surgery), were evaluated.

### 2.1. Subjects

Study group: 28 patients treated for orbital compartment syndrome and retrobulbar hemorrhage underwent orbital decompression surgery as a first-line treatment at our department.

### 2.2. Diagnostics

**Figure 1:**
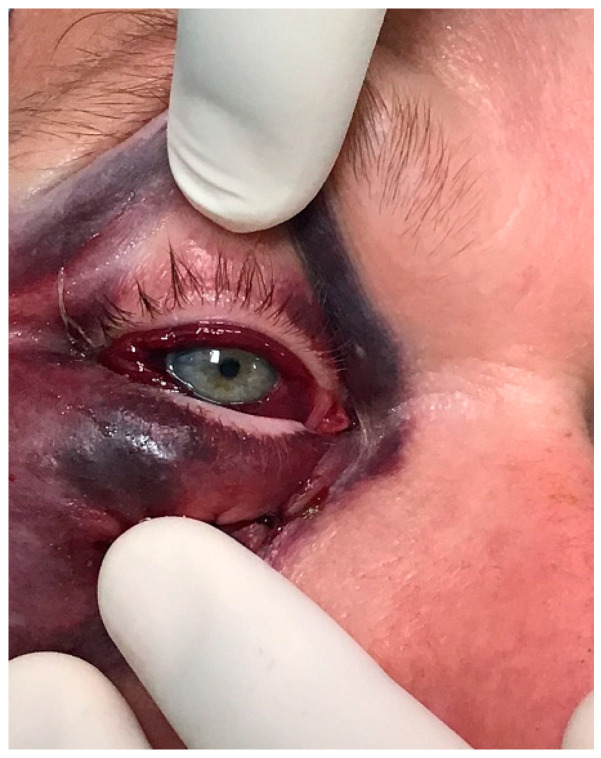
Clinical condition after surgical lipoma removal of right lower eyelid with consecutive postoperative ocular immobility, diplopia, hard eyeball, and impaired visual acuity.

**Figure 2:**
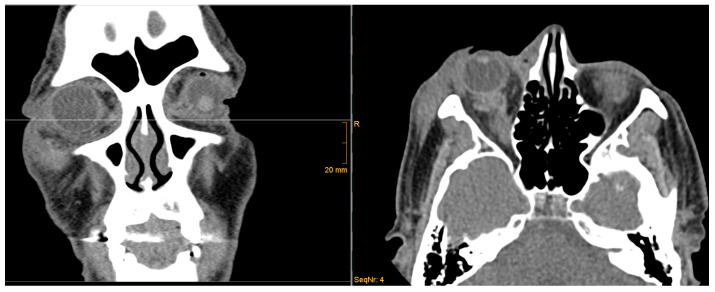
CT scan. Hyperdensity in the right retrobulbar space, swelling of periorbital tissue as evidence of RBH.

The diagnosis of orbital compartment syndrome is based on a clinical examination of the orbit, including impaired visual acuity, ocular immobility, diplopia, a hard eyeball, pain, and proptosis, as well as, if available, CT imaging (see Figure 1 and Figure 2).

OCS symptoms can range from non-present to various symptoms. We defined orbital compartment syndrome as the presence of vision impairment, diplopia, or ocular motility impairment due to orbital pathologic conditions, such as trauma, inflammatory conditions, or orbital tumors, as classified by retrobulbar hemorrhage based on CT imaging. The presence of bleeding, edema, bone displacement, or tumors in the orbit was evaluated in the axial and coronal planes. The elapsed time between the first symptoms of OCS/RBH and surgical treatment, as well as the postinterventional functional outcome regarding visual impairment, was documented.

Visual function was examined pre- and postoperatively (follow-up 24 h) by an ophthalmologist and clinically by an otolaryngologist. VA was measured as in clinical routine and is reported as logMAR and decimal. Visual impairment was classified according to the recommendations of the WHO Consultation on “Development of Standards for Characterization of Vision Loss and Visual Functioning”(see Table 1).

### 2.3. Therapy

All 28 patients received first-line treatment with orbital decompression surgery under general anesthesia. Adjuvant pharmacological therapy was used. Corticosteroids were mostly used. The literature also describes the use of beta-blocker eye drops (Timolol), osmotic diuretic eye drops (Mannitol), and carbonic anhydrase inhibitors (Acetazolamide) to reduce intraocular pressure (IOP).

Orbital decompression surgery aims to reduce pressure on the optic nerve and restore blood flow to the optic nerve and retina, thereby improving visual function. Orbital decompression surgery involves several techniques, often combined depending on the etiology of orbital compartment syndrome (OCS). ODS techniques include lateral decompression (lateral canthotomy), orbital floor decompression, endonasal medial wall decompression (removal of the lamina papyracea), and broad slitting of the periorbital barrier with orbital fat protrusion.

**Lateral wall decompression (LC/IC):** LC/IC can be performed under local anesthesia or general anesthesia. LC is performed via horizontal incision at the lateral canthus. IC is performed via incision of the inferior limb of the lateral canthal tendon to provide prompt pressure reduction of the bulbus. The superior limb of the tendon can also be incised if necessary. If there is no immediate improvement in the patient’s condition or if there are doubts about the results of primary surgical treatment, orbital decompression surgery under general anesthesia must be considered as soon as possible.

**Orbital floor decompression:** transcutaneous approach through incision below the lower eyelid. Partial bone removal of the orbital floor to allow a prolapse of orbital contents into the maxillary sinus to provide pressure reduction.

**Medial wall decompression:** endonasal approach of the medial orbital wall via endoscope or microscope access to the ethmoid sinus. Fractional removal of the lamina papyracea (medial wall) and broad slitting of the periorbital barrier with orbital fat protrusion to allow a prolapse of orbital fat into the ethmoid sinus reducing orbital pressure.

### 2.4. Statistical Analyses

Descriptive analyses were performed first. Continuous variables are expressed as mean and standard deviation, and categorical variables are expressed as frequencies and proportions. Logistic regression analyses were conducted to examine the primary endpoints of postoperative improvement in ocular motility, diplopia, and visual acuity, while accounting for potential risk factors, including age, sex, and time to surgery. The effect measures for all analyses were presented as odds ratios (OR) with 95% confidence intervals (95% CI) and *p*-values (*p*) as appropriate. A *p*-value ≤ 0.05 was considered statistically significant. All analyses were performed using the statistical software SAS 9.4 (SAS Institute Inc., Cary, NC, USA).

## 3. Results

The specializations of first contact upon occurrence of symptoms were 61% in the ophthalmology department, 21% in the Ear, Nose and Throat department (ENT), and 18% in the other specialization departments (internal medicine, trauma surgery, oncology).

### 3.1. Patient Characteristics

The average age range of the patients was 53.18 years (SD: 20.25). A total of 17 patients (61%) were male, while 11 were female (39%).

Evaluation of OCS etiology indicated trauma (centro-lateral midface injury due to a downfall or strike) in 19 out of 28 cases (68%). In four cases (14%), iatrogenic etiology (ophthalmic surgery, sinus surgery) was identified. Tumor occurred in three cases (11%), while inflammatory conditions were found in only two cases (7%).

In 13 cases (46%), the left eye was affected; while in 15 cases (54%), the right eye was affected.

The preoperative best corrected visual acuity was 0.2 (SD: 0.3) on average. Thirteen patients (46%) were preoperatively measured with a visual acuity of 0.05 or less, categorized as ”blindness“ according to the WHO visual impairment categories. A total of 96% (27 cases) showed preoperative ocular motility impairment. Diplopia was preoperatively present in 46% (13) of the cases.

### 3.2. Surgery

A total of 28 patients treated for orbital compartment syndrome underwent orbital decompression surgery as a first-line treatment. Surgery was performed under general anesthesia in all cases.

Orbital decompression surgery was performed with a median of 8.40 h (Q1: 4.8; Q3: 24.0) upon occurrence of symptoms. Eighty-two percent (23 patients) received surgical treatment within 1–24 h after the occurrence of symptoms. Five (18%) patients received therapy after 24 h.

The time between the occurrence of symptoms and surgery, in hours, showed several outliers regarding etiology. Inflammatory etiology (*n* = 2, median [Q1; Q3]: 180.0 [24.0; 336.0]) and tumor etiology (*n* = 3, 48.0 [24.0; 168.0]) revealed high observed times to surgery.

Trauma etiology (*n* = 19, 7.2 [4.8; 12.0]) and iatrogenic etiology (*n* = 4, 3.9 [2.7; 6.0]) showed considerably lower times until decompression surgery than tumor and inflammation etiology.

Medial wall decompression was the most performed decompression technique in 26 out of 28 cases (93%), followed by lateral decompression (LC/IC) in 23 out of 28 cases (82%). Orbital floor wall decompression was performed in only four cases (14%). Surgery techniques were often combined depending on etiology, degree of orbital compartment syndrome, and surgeon experience.

The median time to surgery for LC/IC treatment as emergency therapy was 25.5 h (3.0; 48.0) upon the occurrence of symptoms. In one case, LC/IC was performed after 3.0 h (iatrogenic etiology) and after 48.0 h (tumor etiology).

Out of the thirteen cases with preoperative blindness, one case (8%) received LC/IC treatment as emergency therapy. Out of the six cases with severe visual impairment, one case (17%) received LC/IC treatment as emergency therapy.

Orbital decompression surgery techniques were combined mostly as two-wall decompression in 68% (19/28) of the cases. Trauma etiology represented the majority of two-wall decompression in 74% (14) of the cases. With iatrogenic (16%, three cases) and tumor etiology (20%, two cases), two-wall decompression was performed on rarer conditions.

A three-wall decompression was performed in three (11%) (of the cases. Two out of three cases (67%) with three-wall decompression were traumatic, while one case (33%) was of iatrogenic origin.

A single one-wall decompression was carried out in 21% of the cases (six cases). The etiology was traumatic in three out of six cases (50%). One-wall decompression was performed infrequently in cases of inflammatory etiology (33%, two cases) and tumor etiology (17%, one case).

### 3.3. Analysis of Preoperative Visual Function

#### 3.3.1. Preoperative Ocular Motility

All patients except one (96%) had preoperative ocular motility disorder in the affected eye. For this single case, trauma was the etiology.

All cases (100%) of iatrogenic, inflammatory, and tumor etiology cases suffered from ocular motility impairment. Impaired ocular motility was present in 18 out of the 19 (95%) trauma cases.

#### 3.3.2. Preoperative Diplopia

Diplopia was preoperatively present in 46% (13) of the cases. A total of 54% of the cases were preoperatively not affected by diplopia.

One out of three tumor patients (33%) was affected by diplopia. All patients with inflammatory etiology (100%, two out of two) had diplopia. One out of four patients (25%) with iatrogenic etiology had diplopia preoperatively. A total of 47% of trauma patients (9 out of 19) suffered from diplopia.

The 15 cases not affected by diplopia trauma patients (67%) represented the main etiology group. Iatrogenic (20%) and tumor (13%) etiology were less affected by diplopia.

#### 3.3.3. Preoperative Visual Acuity (VA)

VA was categorized according to the WHO visual impairment categories. A total number of 13 patients (46.4%) were preoperatively measured with a decimal VA of 0.05 or less categorized as “blind”. Six cases (21.4%) presented with a “severe visual impairment”. Preoperative decimal VA was classified as “mild or no visual impairment” in six cases (21.4%). Three cases (10.7%) had “moderate visual impairment”.

The average preoperative measured VA (logMAR) of the affected eye was 1.0.

Tumor etiology (*n* = 3) showed the highest mean preoperative VA (logMAR) of 1.5. All three patients (100%) were already categorized as “blind” preoperatively.

Both patients with inflammation etiology (*n* = 2) indicated the lowest preoperative VA (logMAR) of 0. Both patients (100%) were classified as “mild or no visual impairment” according to the WHO visual impairment categories.

Patients with iatrogenic etiology (*n* = 4) indicated a mean preoperative VA (logMAR) of 1.0. Two patients (50%) with iatrogenic etiology were classified as “blind”. Severe visual impairment was present in one case (25%) with iatrogenic etiology. Classification of “mild or no visual impairment” was also observed in one case (25%) with iatrogenic etiology.

Trauma patients (*n* = 19) had a mean preoperative VA (logMAR) of 1.0. Eight patients (42%) were already preoperatively categorized as “blind” Five patients (26%) with trauma etiology suffered from “severe visual impairment”, “moderate”, and “mild or no visual impairment” were equally represented with three trauma etiology patients (16%) in each group.

## 4. Analysis of Postoperative Visual Function

### 4.1. *Postoperative* Ocular Motility

Preoperative ocular motility impairment was present in 96% of the patients. After ODS, ocular motility impairment was recorded postoperatively in 32% (*n* = 9) of the patients.

Inflammatory etiology was associated with improvement of ocular motility after ODS in 100% of the cases (*n* = 2), iatrogenic etiology in 75% (*n* = 4), and trauma etiology in 68% (*n* = 19). Tumor etiology (*n* = 3) did not benefit from ODS as ocular motility impairment was postoperatively persistent in 100% of the cases.

The results from logistic regression analysis revealed a clinically relevant decreased chance for improvement in ocular motility for females compared to males (OR [95% confidence interval]: 0.28 [0.04; 2.02]). There was no evidence for an association between improvement in ocular motility and age or time to surgery (see Table 2). The logistic regression should be interpreted with caution due to the small cohort size of this study. As shown, there were wide confidence intervals and no significant correlations.

### 4.2. *Postoperative* Diplopia

Preoperative diplopia was present in 46% of the patients. Diplopia improved by 62% after ODS, and diplopia was postoperatively persistent in 18% of the cases (*n* = 5). After ODS, diplopia improved in 100% of inflammatory etiology cases (*n* = 2).

A total of 26% (*n* = 5) of trauma etiology cases improved after ODS, while 21% (*n* = 4) had persistent diplopia after ODS. Trauma etiology cases were in 53% (*n* = 10) of the cases unaffected by diplopia preoperatively.

Iatrogenic etiology improved by 25% (*n* = 1), whilst 75% (*n* = 4) of iatrogenic etiology was unaffected by diplopia pre- and postoperatively.

Tumor etiology cases (*n* = 3) were less likely to be associated with improvement of diplopia after ODS. A total of 67% (*n* = 2) were unaffected by diplopia pre- and postoperative, while one case (33%) was affected, but this did not improve after ODS.

A logistic regression analysis indicates that the chance for diplopia improvement is lower for females compared to males (OR: 0.23 [0.02; 2.68]), while adjusting for age and time to surgery (see Table 2). The logistic regression should be interpreted with caution due to the small cohort size of this study. As shown, there were wide confidence intervals and no significant correlations.

### 4.3. *Postoperative* Visual Acuity (VA)

After orbital decompression surgery, postoperative visual improvement of the affected eye was measured in 36% (10) of the cases.

Of these 10 cases, 90% (9 cases) were of trauma etiology, and 10% (1 case) were of iatrogenic etiology. No visual improvement was reported in 64% (18) of the cases.

Visual acuity of Trauma patients improved after surgery in 47% of the cases (nine cases).

Iatrogenic etiology patients improved after surgery in 25% of the cases (*n* = 4).

Tumor etiology cases did not show VA improvement (+/−0) after surgery. All three patients (100%) were already categorized as ”blind“ preoperatively.

Both inflammatory etiology cases (*n* = 2) did not show visual improvement (+/−0). Notably, the patients with inflammation etiology had the lowest preoperative VA (logMAR) of 0.

No visual improvement was recorded out of the category ”mild or no visual impairment“ (six cases).

**Figure 3:**
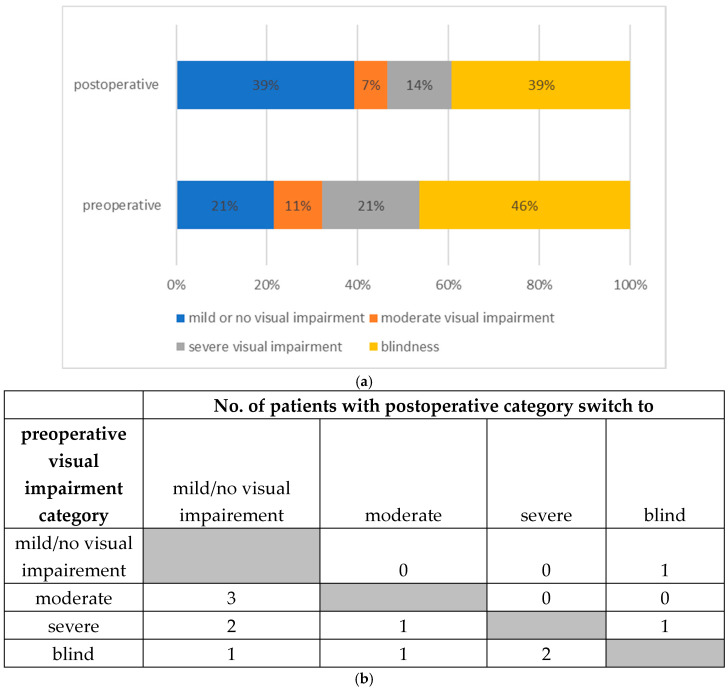
(**a**). Comparison of the WHO visual impairment categories pre-versus postoperatively. (**b**). Individual change of visual impairment categories pre- versus postoperatively.

The group and individual changes of VA are given in Figure 3a,b.

The findings from the logistic regression indicate that females, again, had a lower chance for improvement compared to males (OR: 0.30 [0.04; 2.43]). Furthermore, the chance for improvement decreased with the increasing time to surgery (OR: 0.94 [0.85; 1.04]) (see Table 2). The logistic regression should be interpreted with caution due to the small cohort size of this study. As shown, there were wide confidence intervals and no significant correlations.

## 5. Discussion

In our study, 28 patients treated for orbital compartment syndrome and retrobulbar hemorrhage underwent orbital decompression surgery as a first-line treatment. An evaluation of our study group etiologies identified trauma as the main etiology.

These findings are consistent with the literature. Christie et al. (2018) [13] identified facial trauma etiology and surgery as the main precipitating factors for RBH. Yuen et al. (2003) cited inflammation etiology in up to 6% accountable for orbital disorders [14]. RBH due to tumor etiology is less common, and only a few case reports are documented. Maurer et al. (2013) [15] found OCS mostly in male patients in 68% of the cases, consistent with our study results of 61% in the male population.

The diagnosis of orbital compartment syndrome and retrobulbar hemorrhage with acute visual impairment is considered a primarily clinical diagnosis based on clinical examination underlining the time-critical aspect as an ophthalmic emergency.

Several authors have recommended orbital decompression surgery within 2 h in case of retrobulbar hemorrhage, along with the results from Hayreh and Weingeist (1980) for animal studies of irreversible damage of the optic nerve due to ischemia after 105 min [7,8,16,17].

Comparing time to surgery with the literature shows a wide range of time delays before surgical intervention. Soare et al. (2015) [11] reviewed clinical cases series of orbital decompression surgery. A total of 48% of the listed papers labeled the time to surgery without specifying the exact time data. To some extent case reports with even one case were published [18,19].

Time to surgery varied from less than 1 h to 360 h upon the occurrence of symptoms, consistent with results from our study data; in our study, orbital decompression surgery was performed within a range of 2.4 to 336 h. Most cases (82%) received surgical treatment within 24 h of symptoms, consistent with data published by Christie et al. (2018) [13].

Our study data indicated several outliers regarding the time between the occurrence of symptoms and surgery. Trauma etiology (median 7.2 h) and iatrogenic etiology (median 3.9 h) showed considerably lower times until decompression surgery than tumor (median 48.0 h) and inflammation etiology (median 180.0 h).

Therefore, the late occurrence of symptoms and delayed patient presentation in our department in the case of tumor and inflammation etiology explains the higher time needed for surgery data. Patients with relatively slower symptomatic progression and moderate impact on vision impairment develop rather subacute ODS symptoms [20]. In the case of inflammatory etiology, 100% of our patients presented with a normal decimal VA preoperatively.

According to Yuen et al. (2003) [14], in the case of inflammation etiology, symptoms mainly develop acutely (hours to days) but also occur subacutely over weeks or even over months, explaining a considerably higher time to surgery data. Muscente et al. (2024) indicate, as treatment options of inflammatory orbital compartment syndrome, clinical observation, NSAID, and steroid therapy, which explains a delayed surgical intervention in refractory cases as an acute ophthalmological surgical emergency was initially not the case in inflammatory patients of our study group [21].

Although surgical intervention after the occurrence of symptoms in patients with inflammatory and tumor etiology was relatively late, McCallum et al. (2019) considered orbital decompression surgery as still worthy in case of delayed presentation [22].

Postoperative visual improvement after orbital decompression surgery was measured in 36% of our study cohort patients. Compared to the literature review of Soare et al. (2015) [11] with VA improvement after ODS in 66% of the cases, our results are considerably lower.

The difference in VA improvement of our study cohort might be explained by a relatively longer time to surgery, and the fact that LC/IC under local anesthesia was not commonly used as an emergency decompression technique. Following our study results, improvement in surgical staff training to overcome the reluctance to perform emergency LC/IC was planned.

Furthermore, preoperative blindness is a predictor of persistent blindness despite surgical decompression. Fuller and Hutton (1990) [23] postulated the amount of damage to the optic nerve or macula at the time of injury is the most important prognostic factor. The surgical outcome is, therefore, usually determined by the patient’s visual acuity [23].

A relatively high number of our study patients were already preoperatively severely affected by visual impairment. Preoperatively, 46% of our study patients were categorized as “blind”, or suffered in 26% from “severe visual impairment”, according to the WHO visual impairment categories. These findings are consistent with Maurer et al. (2013) [15], who linked blindness to a retrobulbar hemorrhage in 48% of the patients.

The risk of permanent loss of visual acuity in case of an impaired visual function due to retrobulbar hemorrhage ranges from 48% to 52%, according to the literature; this is consistent with our retrospective data. The number of patients within the VA category for “blindness” dropped after orbital decompression surgery OCS from 46% to 39%.

Orbital decompression surgery techniques were combined mostly as two-wall decompression in 68% of the cases. A single one-wall decompression was carried out in 21% of the cases, while a three-wall decompression was performed in only 11% of the cases. Medial wall decompression was the most performed in 93% of the cases, followed by lateral wall decompression (82%) and orbital floor wall decompression (14%).

It is noteworthy that visual acuity recovery after orbital decompression surgery is not always immediate, according to McCallum et al. (2019) [22]. Our study had a relatively short follow-up period; therefore, it is reasonable that a long-term follow-up might show better visual acuity results.

Preoperative ocular motility impairment was present in 96% of the cases. After orbital decompression surgery, ocular motility impairment was recorded in only 32% of the cases.

These positive results can be interpreted as effective pressure relief to the orbit after ODS. In case of persistent ocular motility impairment after ODS post-surgery, muscular adhesions, secondary scarring, or irreversible muscle fibrosis are possible reasons for non-responders. A short follow-up is noteworthy as bulbus motility was directly examined postoperatively within 24 h. A long-term follow-up may show better postoperative ocular motility results.

Preoperative diplopia was present in 46% of the cases, while after OCS, diplopia was persistent in only 18% of the cases. Equally to ocular motility outcome, these positive results can be interpreted as effective pressure relief to the orbit after ODS. In case of persistent post-surgery diplopia, possible reasons for non-responders are muscular adhesions, secondary scarring, or irreversible muscle fibrosis. A short follow-up is noteworthy as patients were postoperatively examined for diplopia within 24 h. A long-term follow-up may show better postoperative ocular motility results.

Time management of acute retrobulbar hemorrhage diagnosis and treatment must be identified as an essential factor for OCS treatment outcome. From the patients’ first presentation in the emergency department to the correct identification of OCS/RBH, several critical aspects might affect the timing of surgical intervention.

According to Edmunds et al. (2019) [9], 96% of non-ophthalmic emergency department (ED) physicians know the consequences of permanent vision loss due to delayed treatment in the case of orbital compartment syndrome. Although 83% correctly diagnosed RBH in a clinical case survey, only 37% of ED physicians would theoretically perform LC/IC as a first-line treatment due to a lack of training or concerns regarding injuring the patient’s eye.

Compared to our real-life study results, we even identify a considerably higher reluctance towards performing emergency LC/IC—even amongst physicians in ophthalmology and ENT. A fast and precise assessment and therapy of OCS/RBH can be challenging, especially in times of crowded emergency departments and limited access to specialized personnel.

Improvement in surgical training independent from the physicians’ specialties is crucial to overcome the reluctance to perform emergency LC/IC upon a patient’s first presentation to prevent time delay of OCS/RBH treatment in the future.

Due to its time-critical aspect, a CT should not be made before LC/IC in case of suspected RBH. The potential risks of performing immediate LC/IC are manageable and, therefore, should outweigh the high risk of permanent vision loss in case of delayed surgical intervention [24].

Lastly, our study has limitations regarding several cases underlining the rare aspect of OCS and retrobulbar hemorrhage etiology.

The logistic regression should be interpreted with caution due to the small cohort size of this study. As shown, there were wide confidence intervals and no significant correlations. Notably, most of the published literature reports a relatively small number of cases due to the rare occurrence of OCS and retrobulbar hemorrhage etiology.

Although we did not find significant results regarding the timing of surgery and postoperative outcome regarding diplopia, ocular motility impairment, and visual improvement, the consequences of delayed surgical orbital compartment syndrome treatment are assumed to be severe. Preoperative blindness is a predictor of persistent blindness despite surgical decompression.

The most important prognostic factor is the amount of damage to the optic nerve at the time of injury. The surgical outcome is, therefore, usually determined by the patient’s visual acuity. Our study group had a relatively high number of patients severely affected by RBH, reflected by the high rate of patients preoperatively categorized as “blind” or with “severe visual impairment” and a relatively high time to surgery, underlining the time-critical aspect of OCS/RBH treatment.

## 6. Conclusions

Retrobulbar hemorrhage can cause acute visual impairment due to orbital compartment syndrome. Orbital decompression surgery is a valuable time-critical tool in the first-line treatment of acute visual function loss. Our data showed considerable postoperative improvement rates regarding visual acuity, diplopia, and ocular motility, and underlines the need to optimize the time management of acute retrobulbar hemorrhage treatment due to its critical time aspect and immense effect on vision recovery. There is a need to improve the surgical training of (emergency department) physicians, regardless of specialty, to perform LC/IC as emergency therapy.

## Figures and Tables

**Table 1:** The WHO visual impairment categories ICD-10 Version [12].

Category	Presenting Distance Visual Acuity (VA) in Snellen VA ** and Decimal VA ***	
	Worse Than:	Equal to or Better Than:
**0 Mild or no visual impairment**		6/18 3/10 (0.3) 20/70
**1 Moderate visual impairment**	6/18 3/10 (0.3) 20/70	6/60 1/10 (0.1) 20/200
**2 Severe visual impairment**	6/60 1/10 (0.1) 20/200	3/60 1/20 (0.05) 20/400
**3 Blindness**	3/60 1/20 (0.05) 20/400	1/60 * 1/50 (0.02) 5/300 (20/1200)
**4 Blindness**	1/60 * 1/50 (0.02) 5/300 (20/1200)	Light perception
**5 Blindness**	No light perception	
**6**	Undetermined or unspecified	

* or counts fingers (CF) at 1 m. ** Snellen VA. Written as a fraction, like 20/20, 20/40, etc. *** Decimal VA. Ratio of Snellen values.

**Table 2:** Results from logistic regression models.

Covariate	Odds Ratio	95%-Confidence Interval	*p*-Value
**Postoperative Ocular Motility Improvement**			
Age	1.030	[0.983; 1.079]	0.2117
Sex (female vs. male)	0.277	[0.038; 2.019]	0.2054
Time to surgery in hours	1.001	[0.989; 1.013]	0.8947
**Postoperative Diplopia Improvement**			
Age	0.990	[0.946; 1.037]	0.6782
Sex (female vs. male)	0.230	[0.020; 2.680]	0.2407
Time to surgery in hours	1.010	[0.996; 1.025]	0.1572
**Postoperative Visual Acuity Improvement**			
Age	1.006	[0.963; 1.052]	0.7872
Sex (female vs. male)	0.304	[0.038; 2.426]	0.2611
Time in hours	0.938	[0.849; 1.036]	0.2050

## Data Availability

Data available from the corresponding author upon request.
